# No transmission of hepatitis E virus in pigs fed diets containing commercial spray-dried porcine plasma: a retrospective study of samples from several swine trials

**DOI:** 10.1186/s12985-014-0232-x

**Published:** 2014-12-24

**Authors:** Joan Pujols, Carmen Rodríguez, Nuria Navarro, Sonia Pina-Pedrero, Joy M Campbell, Joe Crenshaw, Javier Polo

**Affiliations:** Centre de Recerca en Sanitat Animal (CReSA), Fundación UAB-IRTA, Campus de la Universitat Autònoma de Barcelona, 08193 Cerdanyola del Vallès, Barcelona, Spain; Institut de Recerca i Tecnologia Agroalimentàries (IRTA), Barcelona, Spain; APC EUROPE, S.A. Avda, Sant Julià 246-258, Pol. Ind. El Congost, E-08400 Granollers, Spain; APC Inc., 2425 SE Oak Tree Court, Ankeny, IA 50021 USA

**Keywords:** Antibodies, Hepatitis E virus, Pigs, Spray-dried porcine plasma, Viral genome

## Abstract

**Background:**

Hepatitis E virus (HEV) has been reported in the human population and pigs are a recognized reservoir for HEV and a possible source of HEV transmission to humans. Spray-dried porcine plasma (SDPP) is an ingredient commonly used in feed for pigs around the world. Even though processing conditions used to produce SDPP should be adequate to inactivate HEV, it was of interest to analyze commercial SDPP samples for presence of genome and antibodies (AB) against HEV and to retrospectively analyze serum samples collected from pigs used in past experiments that had been fed diets containing either 0% or 8% SDPP to detect potential transmission of HEV as determined by seroconversion.

**Results:**

Eighty-five commercial SDPP samples were analyzed by ELISA and 100% of them contained AB against HEV, while 22.4% (11 of 49 samples analyzed) were positive for HEV RNA.

Frozen sera samples (n = 140) collected from 70 pigs used in past experiments that had been fed diets containing either 0% or 8% commercial SDPP was analyzed by ELISA for AB against HEV. Age of pigs at sera sampling ranged from 3 to 15 weeks and feeding duration of diets ranged from approximately 4 to 9 weeks. One lot of SDPP used in one experiment was analyzed and confirmed to contain HEV RNA. Regardless of the diet fed, some sera samples collected at the beginning of an experiment contained AB titer against HEV. These sera samples were collected from weaned pigs prior to feeding of the experimental diets and the HEV titer was probably from maternal origin. However, by the end of the experiments, HEV titer was not detected or had declined by more than 50% of the initial titer concentration.

**Conclusions:**

To our knowledge, this is the first study reporting presence of HEV AB titer and RNA in SDPP. Retrospective analysis of serum collected from pigs fed diets with SDPP revealed no indication of seroconversion to HEV. The results indicate that feeding SDPP in diets for pigs does not represent a risk of transmitting HEV, even though HEV genome may be detected in SDPP.

## Resumen

**Antecedentes:**

El virus de la Hepatitis E (HEV) se ha descrito en la población humana y se reconoce a los cerdos como reservorio para el HEV y como posible fuente de transmisión en humanos. El plasma porcino atomizado (SDPP) es un ingrediente comúnmente utilizado en dietas para cerdos a nivel mundial. Aunque el proceso de producción utilizado para el SDPP debería ser suficiente para inactivar el HEV, podría ser de interés analizar muestras comerciales de SDPP para detectar la presencia de material genético y anticuerpos (AB) frente a HEV y retrospectivamente analizar muestras de suero recogidas de cerdos utilizados en estudios anteriores que fueron alimentados con dietas conteniendo tanto 0% o 8% de SDPP para detectar mediante seroconversión la posible transmisión de HEV.

**Resultados:**

Ochenta y ocho muestras comerciales de SDPP fueron analizadas por ELISA y el 100% de ellas contenían AB frente a HEV, mientras que un 22,4% (11 de 49 muestras analizada) eran positivas para ARN de HEV. Muestras de suero congeladas (n = 140) recogidas de 70 cerdos utilizados en experimentos anteriores que habían consumido dietas conteniendo tanto un 0% o un 8% SDPP comercial fueron analizadas por ELISA para AB frente a HEV. La edad de los cerdos en el momento del muestreo era de entre 3 a 15 semanas y la duración de las dietas fue desde aproximadamente 4 a 9 semanas. Un lote de SDPP utilizado en uno de los experimentos fue analizado y confirmado de contener ARN de HEV. Independientemente de las dietas suministradas, algunas muestras de suero contenían títulos de AB frente a HEV al comienzo de los experimentos. Estas muestras de suero fueron recogidas en lechones antes de ser alimentados con las dietas experimentales por lo que los títulos frente a HEV probablemente se debieran a origen materno. Sin embargo, al final de los experimentos, el título frente a HEV o bien no se detectó o se redujo en más de un 50% respecto a la titulación inicial.

**Conclusión:**

Hasta nuestro conocimiento, este es el primer estudio que reporta la presencia de títulos de AB y ARN de HEV en SDPP. Análisis retrospectivo de suero recogido de lechones alimentados con dietas con SDPP revelaron la no seroconversión frente HEV. Los resultados indican que alimentando SDPP en dietas para cerdos no representa un riesgo de transmitir HEV, incluso si el genoma de HEV pudiera estar presente en SDPP.

## Background

Spray-dried porcine plasma (SDPP) as an ingredient in diets for nursery pigs is well recognized to improve growth rate, feed intake, feed efficiency, and to reduce post-weaning diarrhea, mortality, and morbidity [1,2]. In addition, weaned pigs fed diets supplemented with SDPP had reduced intestinal inflammation, mucosal barrier dysfunction, and diarrhea [3].

In recent years, sporadic cases of Hepatitis E virus (HEV) have been reported in the human population of the USA, Europe, and developed countries of the Asian-Pacific region and this virus is now considered an emerging disease [4]. Pigs are recognized as a potential reservoir for HEV [5,6] and as a possible source of HEV transmission to humans [7-9]. The main transmission route for HEV is fecal-oral [10]. The virus has been identified on swine farms in many geographical areas, including the USA and Europe, and the reported prevalence ranges from 22% to 55% [10,11].

Hepatitis E virus is a non-enveloped positive-sense single-stranded RNA virus that is 27–34 nm in diameter and has been classified in the *Hepeviridae* family, genus *Hepevirus* [10]. Currently four distinct genotypes distributed geographically are described. Genotypes 1 and 2 HEV are restricted to humans whereas only genotypes 3 and 4 have been recovered from pigs, humans and others species and are responsible for sporadic cases of HEV in humans. Genotype 3 is found predominantly in Europe, North America and South America [12-14].

Hepatitis E virus is low to moderately resistant to heat and is almost completely inactivated after 1 h of incubation at 60°C to 66°C for all strains tested [15].

Data collected in different European countries show prevalence in weaner pigs ranging from 8% to 30%, between 20% and 44% in growers and 8% to 73% in fatteners [16]. Similarly, the prevalence of HEV in Spain has been reported to range from 20% to 59% and was widely distributed in nearly 100% of investigated swine farms [17-19]. Therefore, prevalence of HEV is high in all age groups of pigs, including pigs at slaughter age, which could still be infected with HEV.

The objectives of the study were to analyze the presence of HEV RNA and antibodies (AB) in commercial samples of SDPP obtained from a Spanish manufacturer and to retrospectively analyze serum samples collected from pigs used in past studies that were fed diets containing 8% SDPP to determine any potential risk of transmission of HEV as indicated by seroconversion in those animals.

## Results

### Presence of AB and HEV RNA in SDPP

**Figure 1:**
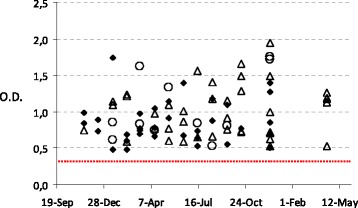
**Antibodies against HEV in 81 different samples of commercial spray-dried porcine plasma batches produced from November 2009 to December 2010.** The established cut-off optical density value was 0.300 for positive detection of antibodies. Each bullet point indicates a Positive (spheres), Negative (triangles) or Unrealized (diamonds) RT-PCR results.

All eighty-five commercial SDPP samples (100%) contained detectable AB against HEV (Figure 1). Eleven of 49 randomly selected samples (22.4%) were RT-PCR positive to HEV genome.

### Retrospective HEV titer analysis of sera samples collected from pigs fed diets containing SDPP

Serum samples (n = 72) from 36 pigs (initial age, 6 weeks) used in an experiment in which pigs were fed diets containing either 0% SDPP (n = 18) or 8% SDPP (n = 18) for 9 weeks was retrospectively analyzed for HEV AB by ELISA. HEV titers were not detected in any serum samples that were collected at day 0 or day 63 of this experiment.

Retrospective HEV titer analysis of a separate set of sera samples (n = 22) collected from 11 pigs (initial age, 3 to 4 weeks) fed diets containing either 0% SDPP (n = 5) or 8% SDPP (n = 6) for 45 days are presented in Table 1. HEV titer was detected in sera from 4 pigs (2 in each group) at the beginning of the experiment; however by the end of the experiment, no HEV titer was detected in any of the samples. Sera samples at the beginning of the experiment were collected prior to feeding experimental diets, so it is probable that the titer detected was of maternal origin. Absence of titer in all sera samples collected at the end of the experiment indicates there was no seroconversion to HEV.

**Table 1:** **Antibody titers against HEV in retained serum samples collected from pigs fed diets with or without spray dried porcine plasma** ^**1**^

**SDPP** ^**2**^	**Pig ID**	**Day 0**	**Day 45**
0%	28	Neg	Neg
0%	1000	Neg	Neg
0%	1027	Neg	Neg
0%	1049	0.752	Neg
0%	1050	1.188	Neg
8%	27	Neg	Neg
8%	999	Neg	Neg
8%	1026	Neg	Neg
8%	1047	1.302	Neg
8%	1048	1.139	Neg
8%	6964	Neg	Neg

^1^Optical density values analyzed by ELISA. Neg means that the O.D. value is below the cut-off established for the technique (<0.300).

^2^Serum collected from pigs weaned at 3 to 4 wks of age and fed diets with either 0% or 8% SDPP for 45 days [33]. Serum samples from pigs were stored at −80°C since the experiment was completed (April to October 2006) and retrospectively analyzed for the presence of HEV AB during April to May 2012.

Results of HEV titer analysis of a third set of sera samples (n = 46) from 23 pigs (initial age, 3.5 weeks) fed diets with 0% SDPP (n = 12) or 8% SDPP (n = 11) for 28 days are presented in Table 2 . Pigs in this experiment were divided into four groups, with two groups challenged with porcine reproductive and respiratory syndrome virus (PRRSV) and fed diets with either 0% SDPP (n = 6) or 8% SDPP (n = 5) or two groups not challenged with PRRSV and fed diets with either 0% SDPP (n = 6) or 8% SDPP (n = 6). HEV titer was detected in serum collected at the beginning of the experiment from 4 pigs (1 pig from each of the 4 groups). By the end of the study (28 days later) only 1 sample that previously contained HEV titer (probably from maternal origin) still had HEV AB, although at a much lower titer (Table 2). The SDPP used in this study was positive for the presence of HEV genome analyzed by nested RT-PCR. The HEV titer results indicate there was no seroconversion, even though the SDPP used in the study contained HEV RNA. Presence of viral genome as determined by PCR does not determine if the genome is capable of causing infection.

**Table 2:** **Antibodies titers against HEV in serum samples from pigs fed diets containing spray dried porcine plasma and challenged with PRRSV** ^1,2^

**SDPP Group** ^**3**^	**PRRSV Challenge** ^**4**^	**Pig ID**	**Day 0**	**Day 28**
8%	Yes	37	Neg	Neg
8%	Yes	40	1.011	0.446
8%	Yes	59	Neg	Neg
8%	Yes	63	Neg	Neg
8%	Yes	64	Neg	Neg
0%	Yes	41	Neg	Neg
0%	Yes	44	Neg	Neg
0%	Yes	46	Neg	Neg
0%	Yes	47	Neg	Neg
0%	Yes	48	0.586	Neg
0%	Yes	57	Neg	Neg
8%	No	68	Neg	Neg
8%	No	70	Neg	Neg
8%	No	72	0.429	Neg
8%	No	75	Neg	Neg
8%	No	78	Neg	Neg
8%	No	79	Neg	Neg
0%	No	84	Neg	Neg
0%	No	87	Neg	Neg
0%	No	88	Neg	Neg
0%	No	97	0.317	Neg
0%	No	98	Neg	Neg
0%	No	99	Neg	Neg

^1^Optical density values analyzed by ELISA. Neg means that the O.D. value is below the cut-off established for the technique (<0.300).

^2^Serum samples from pigs were stored at −80°C since the experiment [34] was completed (April to October, 2006) and retrospectively analyzed for the presence of HEV AB during April to May, 2012.

^3^Respective control or SDPP groups of pigs were fed diets containing 0% or 8% SDPP for 28 days.

^4^Indicated if the pigs were intranasal challenged with PRRSV.

## Discussion

These studies constitute the first survey about the presence of AB against HEV and HEV genome in SDPP. The results indicated that 100% of commercial SDPP samples collected during a 13 month-period contained AB against HEV and 22.4% of samples contained HEV RNA. These results are consistent with the reported HEV AB prevalence of 50% to 100% of pigs at the end of fattening period [17,20], and that 91.5% to 97.6% of farms had pigs with HEV antibodies [19,21]. Likewise, 13.9% of serum samples from pigs older than 6 months were found positive for HEV RNA in a recent Spanish serological survey of 85 farms [18]. Serological studies reported a worldwide distribution of HEV in swine herds located in the USA, New Zealand, Mexico, Japan and European countries [10]. This high percentage of HEV sero-positive SDPP obtained in our study is not surprising, as liquid plasma from approximately 30,000 to 40,000 pigs is pooled to produce a batch of commercial SDPP. Spray dried plasma has previously been shown to contain AB against multiple pathogens circulating in the pig population at any point in time [22]. The presence of AB against HEV in SDPP may have potential to provide passive immunity at the gut mucosal level while being fed to post-weaning pigs. Recent research has demonstrated that liquid porcine plasma contains antibodies against porcine circovirus type 2 (PCV-2) and that after spray drying neutralizing activity was conserved [23].

Under natural conditions, the dynamics of HEV infection is similar to that described for other viral infections in pigs. Acquisition of passive immunity through colostrum absorption (60% of pigs), progressive decline of passive AB at 6 to 12 wk of age, then seroconversion between 14 to 17 wk of age, which is the peak of viremia [10], is followed by a gradual decline to slaughter age [20]. However, this pattern can differ depending on strain of HEV. At Japanese swine farms infected with two common genotype III HEV strains, peak HEV fecal excretion was observed between 1 to 3 mo of age (75% to 100% of the pigs) and by 5 to 6 mo of age, it had declined to 7% of the pigs [24].

Blood is not a primary reservoir of HEV, which is mainly present in liver, stomach, small intestine, spleen, kidneys, salivary glands, tonsils and lungs [10]. However, in Japan it was reported that 10% of pigs at 3 mo of age had HEV in their blood (32/310 positives) but none of the 136 pigs tested positive at 6 mo of age [25]. Similar observations have been reported in a Spanish surveillance study of 6 farrow-to-finish swine herds positive for HEV. Although viremia was observed in some animals at 13 wks of age in one of the herds, none of the pigs at slaughter age from any herd contained HEV in their blood [26]. However, it is possible that pigs with low protective immunity can acquire an HEV infection during their productive life [27] and may contain HEV RNA in blood at slaughter age [26] and as demonstrated in our current analysis of SDPP collected from a Spanish plasma plant. Therefore, although presence of HEV in blood of pigs at slaughter age is low, it is important to demonstrate the absence of HEV transmission risk from feeding pigs diets containing SDPP that may contain HEV RNA.

Heat resistance of HEV is not very high. In cell cultures, HEV was inactivated at 56°C within 30 min or at 66°C during 1 h, depending on virus strain [15,28]. Complete inactivation of HEV in pig liver or in complex meat matrices was achieved at an internal temperature of 71°C [29,30].

Several studies conducted with laboratory spray-driers have demonstrated that the processing conditions used in the plasma industry inactivate low to medium heat resistant viruses like porcine pseudorabies virus (PRV) and PRRSV [31] and even high heat resistant viruses like swine vesicular disease (SVDV) virus [32]. Two recent studies also confirmed that porcine epidemic diarrhea virus (PEDV) was effectively inactivated in plasma by spray drying in a lab drier [33,34]. In addition, several studies have demonstrated that commercial SDPP in diets fed to pigs does not transmit heat resistant viruses such as PCV2 or PPV [31,35-37].

There are numerous features used in the manufacturing process of commercial SDPP that contribute to the bio-safety of this functional protein ingredient. Only blood from healthy pigs that have passed ante-mortem inspection by veterinary competent authorities and approved as fit for slaughter for human consumption is collected for commercially produced SDPP. Avoidance of collecting plasma from clinically affected pigs decreases the risk of potential pathogen transmission; however, in case of asymptomatic diseases like HEV, the safety features of the whole manufacturing process should assure inactivation of such pathogens that cannot be detected at inspection. Other safety features, in addition of the pooling effect mentioned earlier includes spray-drying at high processing temperatures.

Spray drying is the transformation of a feed from a fluid state into a dried particulate by spraying the feed into a gaseous drying medium. The spray-drying process can be divided into 3 significant steps, including atomization of the liquid feed, interaction of the liquid droplet with the drying gases, and separation of the dried powder from the drying gases [38,39]. The conditions in each step can affect the physical characteristics of the powder and microbial survival [39]. The spray drying process used in commercial manufacturing of SDPP has demonstrated its efficacy as a pasteurization-like process to inactivate bacteria and viruses [35] as indicated above. The spray-drying process submits liquid plasma to a thermal process of > 80°C throughout its substance. Therefore, the heat treatment used during the spray-drying process is theoretically adequate to inactivate HEV if present in the raw material. In addition, numerous pathogens do not survive well in a dehydrated substance like SDPP (moisture < 9% and water activity <0.6) that is stored in dry environment for at least 2 weeks prior to release for sale. Furthermore, the inherent neutralizing antibodies in pooled liquid plasma can be regarded as an additional effective safety feature of the manufacturing process for SDPP [23,36]. Recent evidence indicates that neutralizing antibody activity is maintained even after plasma is spray-dried [23]. All these different safety features of the manufacturing process for SDPP (healthy animals, dilution factor, spray-drying process, dry environment, storage at room temperature for at least two weeks and inherent neutralizing antibodies) collectively contribute to the safety of SDPP as a feed ingredient as demonstrated for a variety of swine pathogens previously studied [31,33-37].

Results from our retrospective analysis of serum samples collected from pigs fed commercial SDPP in 3 different experiments indicated absence of HEV virus transmission by feeding diets with SDPP, as observed by the lack of HEV seroconversion. In the results reported in Table 2, HEV seroconversion was not detected even though pigs were experimentally infected with PRRSV, which may make pigs potentially more susceptible to other infections due to the immune depression characteristics of PRRSV infection. A sample of the SDPP used in the diets associated with the experiment reported in Table 2 was PCR positive for HEV RNA; however, no HEV seroconversion was determined in the serum samples of pigs fed the diets with this SDPP lot even though some of these pigs were immune compromised due to PRRSV challenge. Samples of SDPP used in the other experiments were not available, so it was not possible to determine if these samples contained either HEV RNA or titer. However a retrospective serological study conducted in Spain showed that endemic HEV infection in pigs had been present in the Spanish swine population since at least 1985 [17]. Therefore, it can be speculated that the commercial Spanish SDPP used in the experiments likely contained HEV titer and/or RNA. Nevertheless, it should be highlighted that the presence of viral genome analyzed by RT-PCR in SDPP does not indicate infectivity, because this technique is unable to distinguish between infectious and non-infectious virus particles [35,40]. Consequently, the potential infectiveness of SDPP cannot be established by RT-PCR results and studies like the ones reported in this document are needed to determine infectivity potential of viral genome.

## Conclusions

HEV antibodies were detected in 100% of the SDPP samples collected from a Spanish manufacturing plant and 22.4% of these samples also contained HEV RNA, indicating the high prevalence of HEV in the Spanish pig population. In addition, 70 sera samples from 35 pigs from 3 to 15 weeks of age at the beginning of been fed diets containing 8% SDPP for 4 to 9 weeks did not demonstrate seroconversion to HEV. According to the conditions used in this study, the results indicated that feeding SDPP in diets for pigs does not represent a risk of transmitting HEV.

## Materials and methods

### Analytical techniques

#### HEV enzyme linked inmuno-assay (ELISA)

IgG antibodies to HEV in diluted SDPP samples (9% w/v in distilled water) or serum samples collected from pigs fed diets with SDPP in three separate experiments were analyzed using an in-house developed ELISA assay [41]. Briefly, polystyrene plates with 96 wells (Costar 3590) were coated overnight at 4°C with a purified open reading frame 2 truncated protein; HEV-ORF2-6His, the main virus capsid protein from porcine genotype 3 F strain. Samples were added at a dilution of 1:100. To detect pig antibodies to HEV a conjugated HRP anti-porcine IgG secondary antibody was used and TMB was used as a chromogen. Readings were done at 450 nm. A negative and a positive control serum were also analyzed at dilutions of 1:50, 1:100, 1:200, and 1:400. Cut-off was 0.300 O.D. and was determined using four times the SD calculated for control serum.

#### Hepatitis E virus by semi-nested reverse transcription-PCR (RT-PCR)

Viral RNA from diluted SDPP samples was extracted using the Nucleospin® RNA virus kit (Macherey-Nagel Gmbh & Co, Düren, Germany) following the manufacturer’s recommendations. Hepatitis E virus RNA was detected according to a semi-nested RT-PCR developed by De Deus et al. [12].

### Sample collection procedures and storage

#### Presence of AB and HEV RNA in SDPP

Eighty-five spray-dried porcine plasma samples from a Spanish company were collected from 81 different manufacturing batches produced from November 2009 through December 2010. Dried samples were diluted in PBS at a ratio of 1:9 before being analyzed for presence of total AB against HEV by ELISA as previously described. Forty-nine of these samples were selected at random and analyzed for HEV RNA as previously described.

#### Presence of AB in serum samples

Serum samples (n = 72) collected from 36 pigs at 6 and 15 weeks of age that were fed diets with either 0% SDPP (n = 18 pigs) or 8% SDPP (n = 18 pigs) for 9 weeks [31] were retrospectively investigated for the presence of AB against HEV by ELISA. Briefly, these pigs were weaned at 4 wks of age and fed a common diet for 2 wks and determined to be negative for antibodies against PRV, PRRSV and PPV. Subsequently, pigs were allotted to six pens with six pigs per pen and fed diets containing either 0 or 8% SDPP (18 pigs and 3 pens per diet) for 9 wks. Blood samples had been collected from pigs at the beginning and end of the 9 week feeding period to determine whether feeding SDPP caused seroconversion and development of AB against PPV, PRRSV, or PRV.

The blood sampling was conducted from April 4 to June 26, 2000 and HEV analysis was performed from April 4 to May 30, 2012. Serum samples had been maintained at −20°C since the study; however a sample of the SDPP used in the feed had not been retained. Since seroconversion was not detected then PCR analysis was not done.

Serum samples collected from pigs used in a study published by Pujols et al. [35] were retrospectively investigated for the presence of AB against HEV by ELISA. Briefly, the study was conducted to determine whether feeding diets with SDPP containing 2.47 x 10^5^ DNA copies of porcine circovirus type 2 (PCV2) could infect weanling pigs. The two groups of pigs were housed in separate bio-safety level-3 rooms. None of the pigs in either group developed any clinical signs or became PCV2 viraemic or seroconverted.

The blood sampling was conducted from October 9 to December 4, 2006 and the HEV analysis was performed from April 19 to May 30, 2012. Serum samples had been maintained at −80°C since the study. Due to the lack of retrospectively available samples of the SDPP used in the feed (AP820P lot # Y617932) it was not possible to analyze the SDPP for presence of HEV genome.

A third set of serum samples (n = 46) collected from 23 pigs (initial age, 3.5 wks) fed diets containing either 0% SDPP (n = 12 pigs) or 8% SDPP (n = 11 pigs) for 4 wks [36] were retrospectively analyzed for the presence of AB against HEV by ELISA. Briefly, the objective of the experiment was to evaluate if SDPP containing PCV2 genome supplemented in feed could transmit PCV2 to pigs challenged with PRRSV. Twenty-three PRRSV-free pigs at 25 d of age, were housed in bio-safety level 3 facilities and assigned to four groups in a 2x2 factorial design consisting of pigs subjected or not to PRRSV challenge and fed the diets containing either 0% SDPP or 8% SDPP. Challenge groups were inoculated intra-nasally with 2 mL of a suspension containing 10^6^ TCID_50_ PRRSV/mL. Drinking water for pigs fed the diet with 8% SDPP was supplemented from day −4 to day 7 post-inoculation with spray dried porcine serum (SDPS) to deliver a final solution of 2% w/v. Dietary treatments were fed for 28 d post-inoculation (PI). All challenged pigs developed PRRSV viraemia by d 3 PI and PRRSV AB were detected in sera by d 14 PI, with no difference between dietary treatments. Neither PRRSV viraemia nor seroconversion was detected in non-challenged pigs. Porcine circovirus type 2 DNA was not detected in the serum of any pigs throughout the experimental period. Spray dried porcine plasma containing the PCV2 genome supplemented in feed did not result in PCV2 transmission to either healthy or PRRSV-infected pigs under these experimental conditions.

The blood sampling was conducted from March 16 to April 16, 2009 and HEV analysis was performed from April 19 to May 30, 2012. A sample of SDPP used in the feed and serum samples had been maintained at −80°C until analyzed for the presence of RNA HEV genome and AB against HEV.

## Abbreviations

AB: Antibodies
HEV: Hepatitis E virus
SDPP: Spray-dried porcine plasma
PRRSV: Porcine reproductive and respiratory syndrome
PEDV: Porcine epidemic diarrhea virus
PRV: Pseudorabies virus
PCV-2: Porcine circovirus type 2
PPV: Porcine parvovirus
SVDV: Swine vesicular disease virus
